# DEM modelling of surface indentations caused by granular materials: application to wheel–rail sanding

**DOI:** 10.1007/s40571-024-00816-w

**Published:** 2024-09-06

**Authors:** Bettina Suhr, William A. Skipper, Roger Lewis, Klaus Six

**Affiliations:** 1 Virtual Vehicle Research GmbH, Inffeldgasse 21A, 8010 Graz, Austria; 2 Department of Mechanical Engineering, The University of Sheffield, Mappin Street, S1 3JD Sheffield, UK

## Abstract

The presented surface indentation model is one step towards building a DEM model for wheel–rail sanding. In railways, so-called low-adhesion conditions can cause problems in traction and braking, and sanding is used to overcome this problem. Sand grains are blasted towards wheel–rail contact, fracture repeatedly as they enter the nip and are drawn into the contact and then increase adhesion. Research on this topic has mostly been experimental, but focussed on adhesion enhancement measurement. Thus, physical mechanisms increasing the adhesion are not well understood. Previous works involved experiments and DEM modelling of single sand grain crushing tests under realistic wheel–rail contact pressures of 900 MPa, focusing on sand fragment spread and formation of clusters of solidified fragments. In the experiments, indents in the compressing steel plates were also observed, which are also observed on wheel and rail surfaces in railway operation. These are now modelled by adapting an existing surface indentation model from literature to the case of surface indentations caused by granular materials. Two test cases are studied, and experimental spherical indentation tests for model parametrisation are presented. In a proof of concept, the mentioned single sand grain crushing tests under 900 MPa pressure are simulated including the surface indentation model. This work contributes to DEM modelling of wheel–rail sanding, which is believed to be a good approach to deepen the understanding of adhesion increasing mechanisms under sanded conditions.

## Introduction

The motivation for this work is to develop a DEM model for sanded wheel–rail contacts. In railways, sanding of wheel–rail contacts has been used for several decades to overcome so-called low-adhesion conditions. Low adhesion, i.e. an adhesion coefficient below 0.1, negatively influences traction and braking behaviour of railway vehicles in service and can cause safety issues in the worst case [1, 31]. The maximal adhesion coefficient (AC) limits the transferable tangential force in the contact. In general, the wheel–rail contact is characterised by extremely high normal contact stresses, with a maximum in the range of 1 GPa and higher, accompanied by extremely high tangential stresses. The contact condition has a large influence on the AC. Under dry conditions, the AC is around 0.35 or higher [17, 18]. Under some contact conditions, low adhesion occurs, e.g.  damp (wet) contact conditions [30], (‘wet rail’ phenomenon) or when the rail surface is contaminated with leaves [1, 26].

**Fig. 1:**
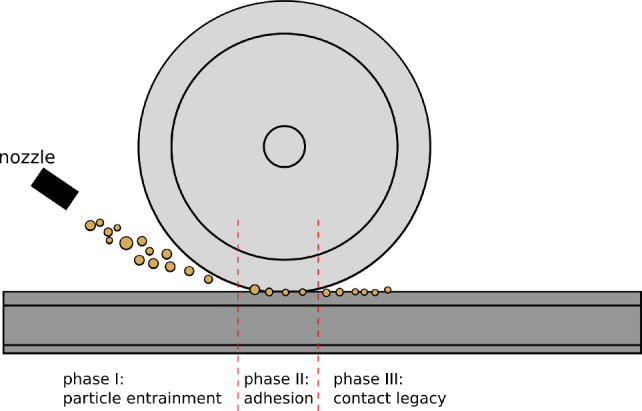
Scheme of wheel–rail contact sanding [23]

Under low-adhesion conditions, the AC can be increased by spraying sand from a nozzle towards the wheel–rail contact, see Fig. 1. Some particles are expelled and some are entrained into the contact. The entrained particles fracture repeatedly and influence the adhesion, also changing the roughness of wheel and rail. While sanding does increase the AC under low-adhesion conditions, it can also lead to increased damage on both wheel and rail [6, 7].

**Fig. 2:**
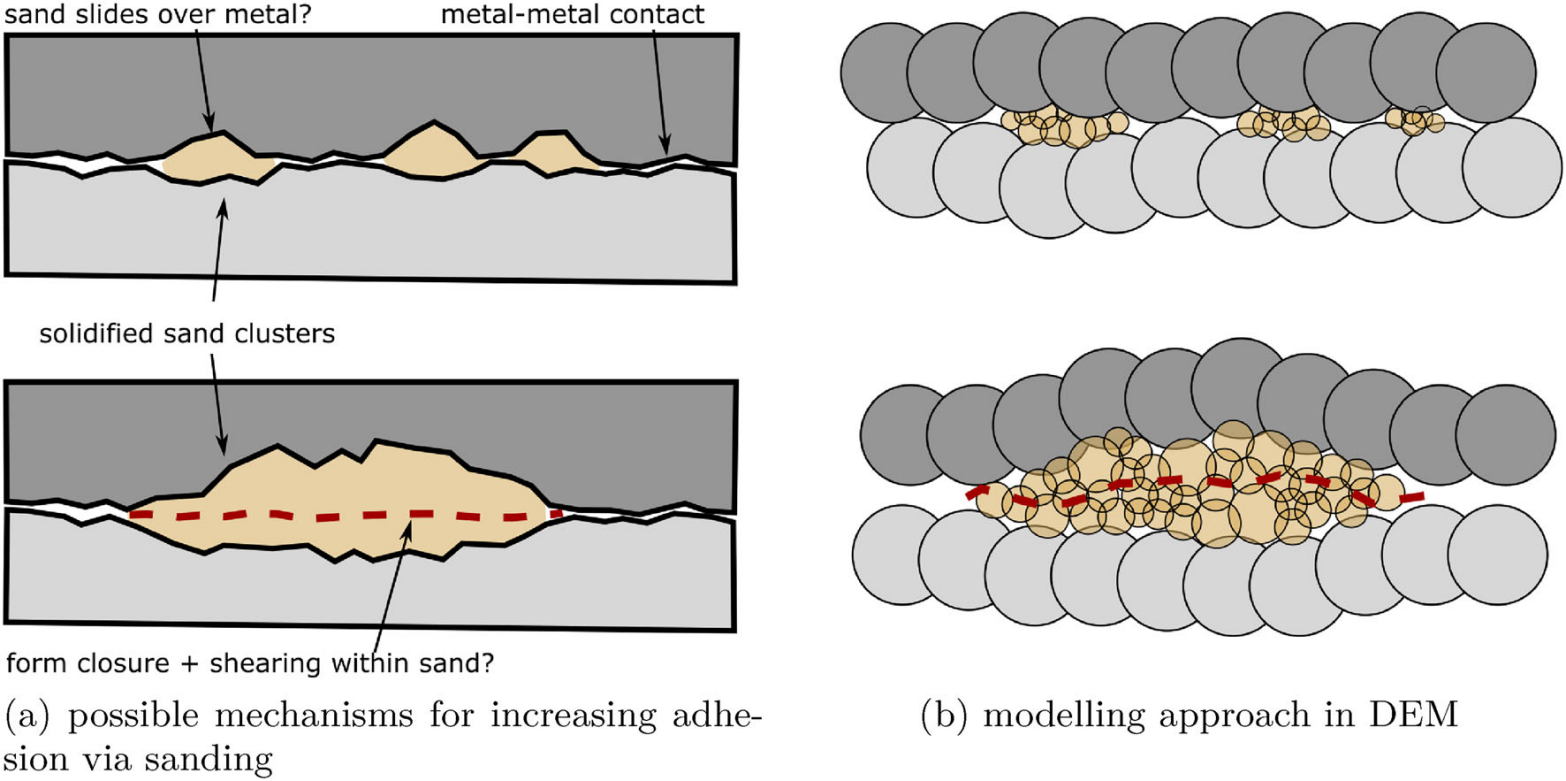
Sanded wheel–rail contact

Wheel–rail sanding is a field of active, but almost purely experimental, research [19, 20], e.g. measuring adhesion coefficients (ACs) under different contact conditions (dry, wet, ...) applying different sands or other particles. In general, modelling of the wheel–rail contact is a wide field of research in tribology, see [4, 8, 10, 22, 27, 29] for review articles as well as recent works. In particular, research works on developing numerical models considering local effects in the wheel–rail contact region during sanding are very sparse and focus on electrical isolation [3, 33], or particle entrainment efficiency [5, 9].

Despite the active research in this field, the physical mechanisms causing the change in ACs under sanded conditions are not yet well understood. Simulation models can help to increase this understanding, when they include the relevant features of the sanding process. When entering the contact, sand grains will fracture repeatedly and some of their fragments will be expelled. The amount of sand in the contact determines whether the metal surfaces are (partially) separated or not, allowing for different mechanisms of load transfer, see left part of Fig. 2. Under high loads, sand fragments solidify and form clusters, which indent into wheel and rail surfaces (affecting roughness) [23]. This could increase adhesion via form closure effects, or the sand powder could solidify and partially cover the rough wheel–rail surfaces, increasing the effective contact area and thereby increasing the AC. The role of water is also unclear.

Two very recent and very different approaches aim at modelling the wheel–rail sanding process. In [32], adhesion enhancement in sanded wheel–rail contact is simulated using a 2D FEM model. The sand grains are modelled as triangles connected by so-called Cohesive Interface Elements allowing for particle breakage. Sanding under traction and braking conditions for differently sized sand grains of circular or ellipsoidal shape are simulated. The wheel–rail contact is set to be frictionless and only contacts involving the sand fragments are frictional with a coefficient of friction of 0.5. As a result, the model does not compute adhesion, but the adhesion enhancement caused by frictional contacts and interlocking of sand fragments. In traction simulations, this adhesion enhancement increased with increasing number of sand fragments and adhesion was generally higher than under braking conditions. In braking simulations, more elongated particles gave the highest adhesion enhancement. Under traction conditions, sand fragments passed the wheel–rail contact, while under braking conditions they were pushed to the end. These effects qualitatively matched observations made from experiments. For comparison of adhesion, no experimental data were available.

In [23, 24], the authors of this study started to build a DEM model of wheel–rail sanding. As a preparation, single sand grain crushing tests of two types of rail sands under dry and wet contact conditions were conducted, [23]. In initial breakage tests and tests under a realistic wheel–rail load of 900 MPa sand fragments spreading behaviour was studied to understand what amount of sand fragments might be expelled from wheel–rail contact and what amount might stay inside and influence adhesion. One type of rail sand used in Great Britain, called GB sand, showed high fragment spread under dry conditions and low spread under wet conditions. On the contrary, rail sand used in Austria, called AT sand, showed low fragment spread both under dry and wet conditions. In the high-loading tests under wet conditions for both rail sands, large clusters of solidified sand fragments formed. High-resolution 3D scans of these sand clusters and the supporting steel plate, showed indents in the steel plates caused by sand clusters in prior tests. This confirms that form closure effects depicted schematically in Fig. 2 might play a role in adhesion increase. In [24], a DEM model of the conducted sand crushing tests was developed and parametrised. For both types of rail sand and both dry and wet contact conditions, a good agreement between experiments and simulations was achieved regarding the sand fragment spread and formation of clusters. As a first step, in [24], the steel plates were modelled as undeformable plate objects.

This work presents the next step, where the steel plates will be represented as plastically indentable surfaces. The modelling approach is sketched in Fig. 2b.

In general, the steel plates could be modelled as elasto-plastic solids using the Finite Element Method in a coupled DEM-FEM approach. Such couplings are used in numerous applications in geomechanics, compare [11–14, 25, 28] to name only some. The main reason to use DEM for modelling the solid’s surface is the possible future use of this model. It allows extending the model with additional physical effects, which are easier modelled with DEM than with continuum methods such as FEM. One important model extension would be the consideration of so-called third body layers (3BL). Such layers consist for example of sand fragments or sand powder clusters, wear debris detaching from wheel and rail surfaces, and other naturally existing or artificially introduced substances. 3BL interact with rough wheel and rail surfaces and affect the frictional behaviour of them. The wear process itself can be included in a numerical model, by considering the initiation and the further propagation of multiple cracks leading to the detachment of wear particles becoming part of the 3BL. Such processes would be hard to include in a (X)FEM or coupled FEM-DEM model but are easier implemented in a pure DEM model.

Works in the literature that model surface wear in DEM are rare. The studies of Pham-Ba and Molinari [15, 16] work at a very small length scale: With roots in Molecular dynamics (MD), a coarse-graining was applied that achieved to use particles of the size of 10 times the atoms they replaced. While these models have considerably lower computational costs than using MD directly, still their computational costs are too high for the aimed application of wheel–rail sanding.

The work of Capozza and Hanley [2], was an ideal starting point for this study. In [2], a surface indentation model was developed, where plastic wear of a surface caused by an indenter sphere was studied. The surface was modelled by a regular hexagonal grid of non-overlapping spheres, the indenter sphere meets the surface under normal or oblique impact or under scratching conditions. When the stress at a surface sphere is higher than its given hardness *H*, then the surface sphere is moved, similar to an ideal plastic material law. The surface spheres are always displaced vertically, i.e. normal to the surface plane, to maintain the regularity of the surface grid. The developed model has a rather low computational effort and as it contains only one parameter, *H*, it is easy to parametrise.

However, the model is formulated for indenter spheres being larger than the surface grid spheres. In this case, there is only one contact between the indenting sphere(s) and each surface sphere. In contrast, when the surface is indented by a granular material, a surface sphere can be in contact with several spheres from the granulate.

In this work, the model from [2] will be extended such that the surface can be indented by a granular material under vertical loading. As an additional novel aspect, this work systematically investigates the influence of the surface grid’s properties in two test cases with purely elastic and elastic-(ideal)-plastic behaviour. Finally, the application of surface indentation under a crushing sand grain at wheel–rail load is new.

This paper is organised as follows: In Sect. 2, the original surface indentation model from [2] is summarised and adaptions for indentations by granular materials are presented. In the following section, the influence of the surface grid is discussed for two test cases: the normal impact of a sphere on the surface grid and the normal compression of a granular material constrained by side walls on the surface grid. For both test cases, the purely elastic behaviour is compared to the elastic-(ideal)plastic behaviour leading to surface indentations. Section 4 contains experimental results of spherical indentation tests on a commonly used rail steel. These results are used to parametrise the hardness of the steel plates in the adapted surface indentation model. The parametrised DEM model is then used in Sect. 5 for a proof of concept: combining the surface indentation model and the previously developed model for sand breakage, single sand grain crushing under realistic wheel–rail load is simulated. Finally, the last section contains conclusions and an outlook on future work.

## Adapted surface indentation model

To consider the experimentally observed plasticity effects on the upper and lower plates in the high-loading sand crushing tests, i.e. development of indentations/ roughness, the surface indentation model presented in [2] has been taken as a starting point and adapted for pure vertical loading by a granular material.

For convenience, the model published in [2] will be summarised first and then the adapted model will be presented. As already mentioned, the work described in [2], studied plastic wear of a surface by an indenter sphere and considered shallow indentations. The developed model could simulate surface indentations under normal or oblique impacts as well as scratching of a surface. The surface was modelled by a regular hexagonal grid of *N* spheres of radius $r_s$. The surface had the area *A* and thus the area of each surface sphere was given by $a_s=A/N$. The surface was assigned the hardness *H* and it was indented by an indenter sphere of radius $R_p$. The indenter’s deformation was neglected because the indenter’s hardness was assumed to be higher than that of the surface. The indentation depth *h* was restricted to $h \ll R_p$ such that surface cracks could be neglected. Contacts between surface spheres were ignored and contacts between the indenter sphere and a surface sphere used the Hertz–Mindlin contact law. The resulting contact force was used to calculate the pressure on the surface (for each surface sphere separately). If the pressure was below the surface’s hardness, the surface remained unchanged and thus the surface spheres did not move. For pressures above the hardness, the positions of surface spheres were adapted to represent the indentations occurring. In this model, the surface spheres were displaced only vertically, i.e. in *z*-direction. The surface spheres were not displaced laterally to ensure the regularity of the surface grid. Clearly, when a surface is indented, its area increases. Thus, when a surface sphere *i* was moved to model the indentation, also its area $a_i$ increased, see [2] for a detailed explanation and a graphical illustration. The equations of the described model are summarised in the box below.
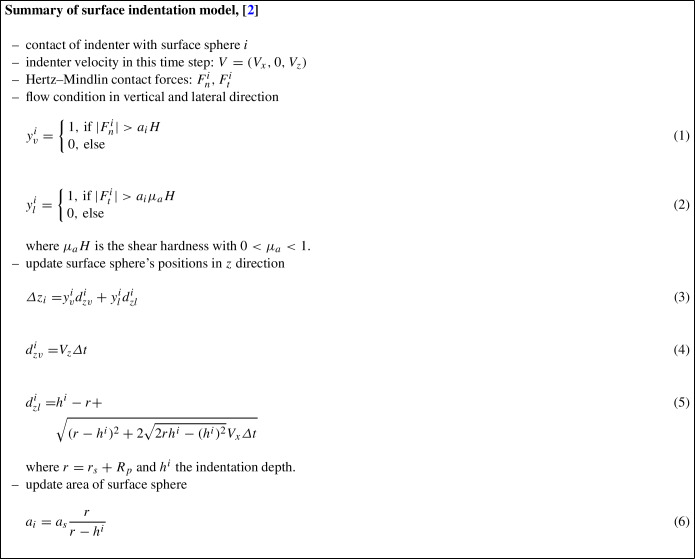


**Fig. 3:**
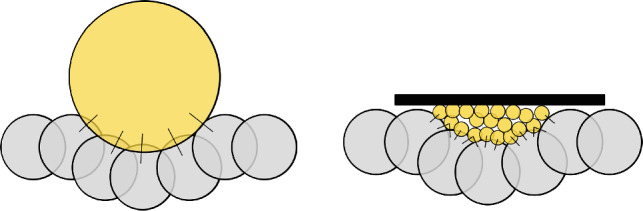
Schematic visualisation of contact situations. Left: large indenter contacting surface grid, one contact per surface sphere. Right: granular material contacting surface grid, multiple contacts per surface sphere

The model described above considers one indenter in contact with the discretised surface. Therefore, each surface sphere has only one contact with the indenter. In contrast, when the surface is indented by a granular material, a surface sphere can be in contact with several spheres from the granulate. Both situations are visualised in the drawing of Fig. 3. The surface indentation model is adapted to take into account multiple contacts. At first, all contact forces are calculated using the Hertz–Mindlin contact law. This includes contacts inside the granular material and contacts between the granular material and surface spheres. Contacts between surface spheres are again ignored. For the flow condition, all *K* contact forces (normal and tangential) of a surface sphere *i* are summed up vectorially. In this work, the surface is oriented perpendicular to the *z*-axis. Therefore, the *z*-component of this sum is used to calculate the pressure acting on this surface sphere. If the flow condition is met, then the surface sphere is displaced. To do so, the relative velocities between the surface sphere and all *K* contacting spheres are calculated. The maximum of the *z*-component of these relative velocities is used to calculate the displacement distance analogously as in the original model. Alternatively, it would also be possible to use, for example, the mean value of the relative velocities. However, the use of the maximum of the relative velocities prevents the “fastest” granular sphere from entering too deeply into the surface sphere. For the small indentation depth considered in this work, the increase of surface area as formulated in [2] is negligible. Therefore, the area of the surface spheres is constant in the adapted model. As the aimed application of the model involves the application of normal forces only, the tangential direction of the indentation model is not considered here. The adapted model is summarised in the box below:

## Test cases and surface grid influence

In this section, two test cases will be studied together with the influence of the used surface grid’s properties. These grids are used to model a solid’s surface. Thus, they are the numerical discretisation of a continuous body and they also introduce a certain surface roughness by their geometric description. While [2] focused on model development using only one surface grid of non-overlapping spheres, the influence of different surface grids should be investigated due to the before mentioned reasons. At first, several surface grids will be constructed and their characteristics will be shown. These grids will be used in all considered test cases. The first test case is the normal impact of a large sphere on the surface, both the purely elastic response and the plastic response using the adapted surface indentation model will be considered. The elastic–plastic case allows for a comparison with the results of [2], checking the influence of the changes made to the model and the use of overlapping surface grids. The second test case will be the normal compression of a granular material on the different surface grids. Again, the purely elastic and the plastic behaviour will be studied.

For all DEM simulations in this work, the software YADE [21] was used. It is open source and utilises the soft contact approach together with explicit integration in time.
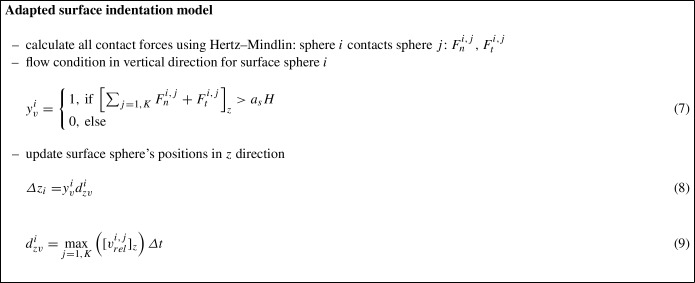

**Fig. 4:**
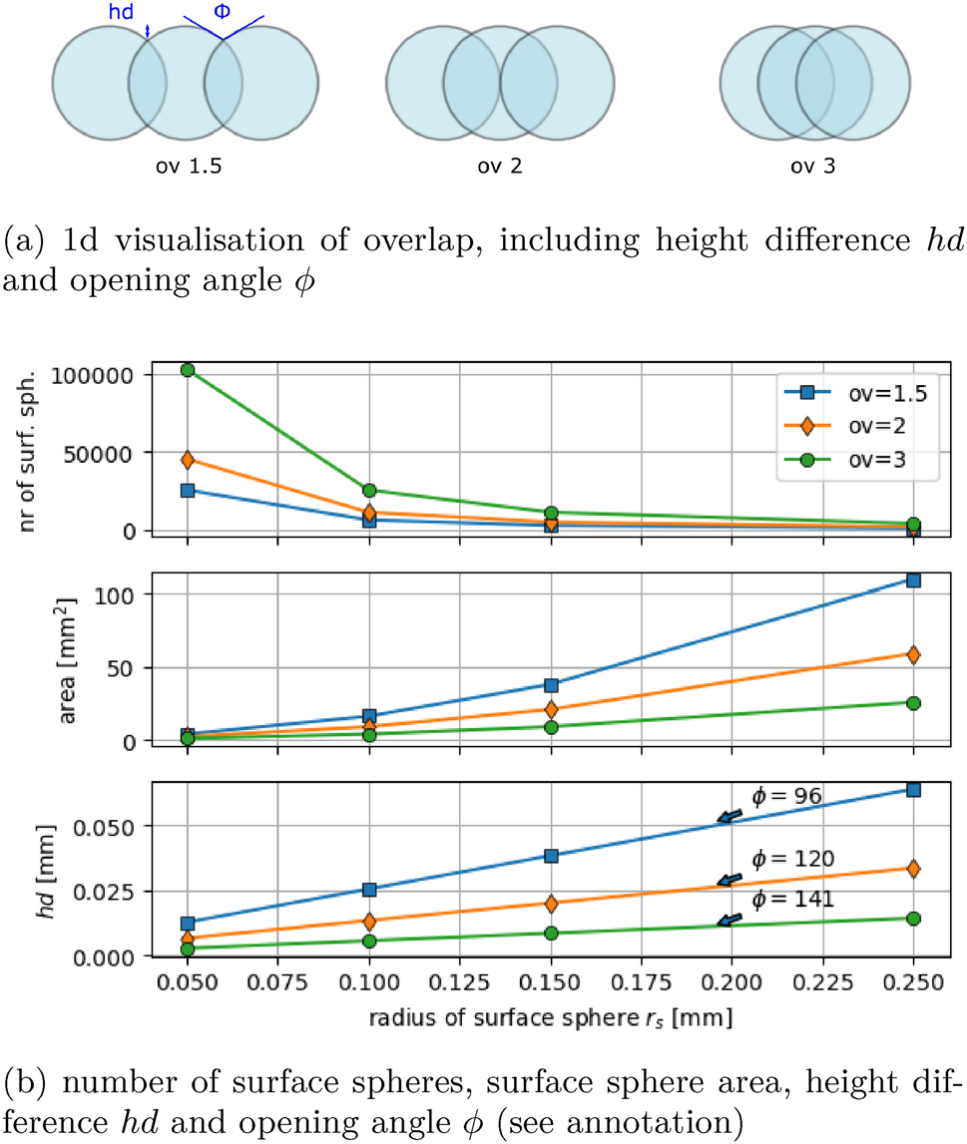
Properties of the constructed surface grids using four different radii and three overlap factors (*ov*)

### Surface grid construction

The used surface grids are regular hexagonal grids of spheres generated with Yade’s grid generator [21]. The function yade.pack.regularHexa() is used to generate grids of touching spheres of a given radius. Grids of touching, i.e. non-overlapping, spheres are considered too rough for approximating a smooth surface. The surface representation gets smoother, when the grid spheres overlap, i.e. the spheres are grown with the multiplicative overlap factor (*ov*) after the generation. A visualisation of the overlap factor can be seen in Fig. 4a for a 1D drawing. This figure also shows the two studied grid properties: the height difference *hd* and the opening angle $\phi$ of the grid.

In this work, only one layer of spheres is generated and the dimensions used are length=width=1 cm. The constructed grids have four different radii of the surface spheres, $r_s= 0.05, 0.1, 0.15, 0.15~\hbox {mm}$, and three different overlap factors, $ov=1.5, 2,3$. Figure 4b shows the properties of the 12 constructed grids. The first subplot shows the number of spheres in the grid, *N*, which increases with increasing *ov* and decreasing $r_s$. The middle subplot shows the area per surface sphere $a_s=1~\text{ cm}^2/\textrm{N}$. The lowest subplot shows the height difference *hd*. It decreases with increasing *ov* and decreasing $r_s$. The opening angle $\phi$ depends only on *ov* (and is independent of $r_s$). Its values are stated in the lowest subplot of Fig. 4b.

### Elastic normal impact of indenter sphere

**Fig. 5:**
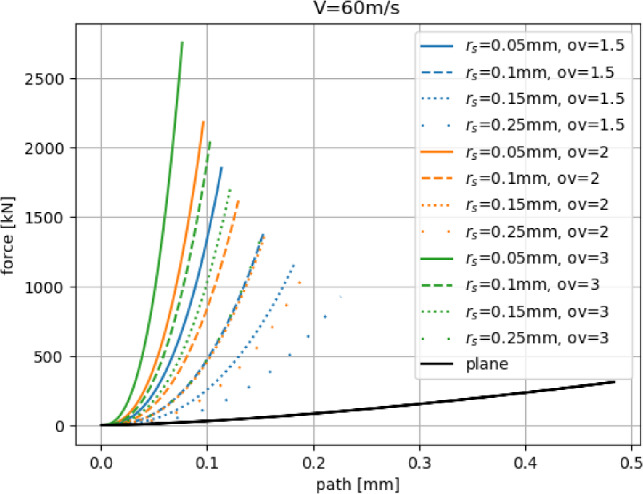
Path-force response in normal indent simulations using different surface grids

In this subsection, the elastic normal impact of an indenter sphere on the constructed surface grids is studied. The indenter radius is $R_p=1~\hbox {cm}$, and the contact parameters of the Hertz–Mindlin model are given in Table 1. The indenter sphere approaches the surface with $V=60~\hbox {m/s}$ and the resulting path-force curves are shown in Fig. 5. Results for a simulation where the indenter sphere impacts a plane are also shown (according to Hertzian theory). The surface grids represent rough surfaces rather than the smooth surface of the plane. Initially, the indenter is in contact with only one surface sphere, and the equivalent radius, $R^*$, in the Hertz contact law is approximately equal to $r_s$. On the contrary, when the indenter contacts the plane, $R^*=R_p$. Initially, the resulting force is lower in the case of indenter and surface grid compared to indenter and plane. This changes soon, as the indenter gets into contact with additional surface spheres. The number of contacting spheres depends on the surface sphere radius $r_s$ and the overlap factor *ov*. Over the complete impact simulation, the response of surface grids is much stiffer compared to the plane case, which of course does not make sense physically because, in reality, plastic deformations would occur locally reducing the overall stiffness. This will become clearer in the next subsection, where the elastic–plastic behaviour of the surface is studied.

**Table 1:** Parameters of Hertz–Mindlin contact law equally used for indenter sphere and surface spheres

*E* [GPa]	$\nu$ [-]	$\mu$ [-]	$\rho \left[ \frac{\text{ kg }}{\text{ m}^3}\right]$
400	0.3	0.5	8000

### Plastic normal impact of indenter sphere

In this subsection, the plastic indentation of the surface is studied when impacted by the indenter sphere. Due to the choice of $R_p$ and the material parameters of Table 1, the results can be directly compared to those obtained in [2], Sec. 4.1, where a grid of non-overlapping spheres was used. Also, a theoretical estimation of the indented volume for this test case was derived. Neglecting the effect of elastic deformation, in [2] the following estimation was obtained:

10 $$\begin{aligned} I_V^{th}=\frac{V^2 m}{2 H}, \end{aligned}$$

where *V* denotes the indenter’s initial velocity, *m* its mass, and *H* its hardness. The corresponding indentation volume, $I_V$, can be calculated from simulations using the positions of the surface spheres:

11 $$\begin{aligned} I_V (t)=\sum _{i=1}^N \left( z_i(0) - z_i(t)\right) a_s, \end{aligned}$$

where $z_i(t)$ is the *z*-coordinate of surface sphere *i* at time *t*.

The available results from both theory and simulation allow to investigate the influence of the surface grid as well as the influence of the changes made to the model, i.e. adapted computation of the flow condition and the neglect of the increase in surface area.

**Fig. 6:**
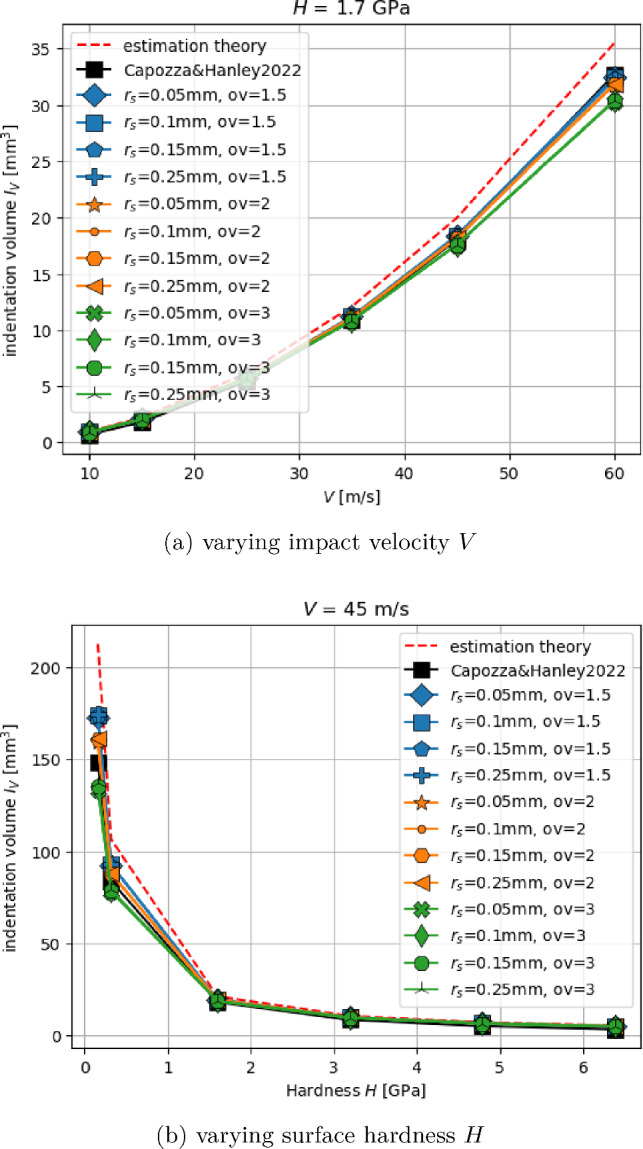
Indentation volume $I_V$ resulting from normal impact with different velocities *V* and surface hardness *H*

Figure 6 shows indentation volumes for varying *V* and constant surface hardness $H=1.7~\hbox {GPa}$, Fig. 6a, and for constant $V=45~\hbox {m/s}$ and varying *H*, Fig. 6b. Simulated indentation volumes are plotted, calculated by eq. (11), for all 12 surface grids. For comparison, also the estimated indentation volume calculated by eq. (10) and the simulation results obtained in [2] (using a non-overlapping surface grid) are given. The results show similar trends in both cases of Fig. 6. For smaller indentation volumes, $I_V<20 \text{ mm}^3$, all surface grids give similar results, and the results are in good accordance with the results from [2] and theoretical estimation. For larger indentation volumes, differences depending on the surface grid can be seen. While the agreement with the simulations of [2] is still given, the results are now lower than the theoretical estimation. The observed differences between the surface grids depend only on the overlap factor *ov* and not on $r_s$. With increasing *ov* values, the simulated $I_V$ decreases. In this test case, there is only one contact per surface sphere with the indenter. Thus, the flow condition simplifies to $\left[ F_n \right] _z>a_s H$. The computation of $F_n$ involves only the surface sphere’s radius $r_s$ and is independent of *ov*. On the contrary, the calculation of $a_s$ involves both $r_s$ and *ov*, which explains the dependency of the results on *ov*. Despite the differences that occur, it is important to note that for small to moderate indentation volumes, little differences can be seen between the 12 surface grids. From the adaptations made in the model, two are relevant in this test case: in the flow condition, the *z*-component of the contact force is used, and the increase of the surface spheres’ area is neglected. Due to the good agreement of the results with those of [2], both changes are justified.

### Elastic normal compression of granular material

**Fig. 7:**
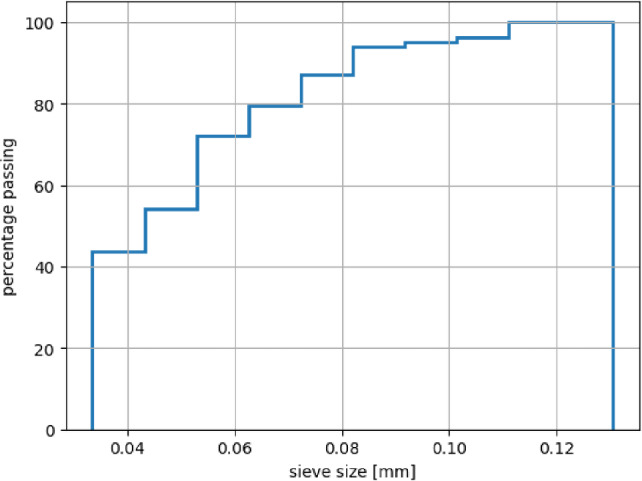
Particle size distribution curve of granular material used in simulations

In this subsection, a granular material is studied under normal compression on the surface grids. As a first step, only the elastic behaviour is studied and compared to the case of compression on an ideal plane. The intended application of this model is the indentation of sand grain fragments on the steel surface, as in sanded wheel–rail contacts. Therefore, information from previous works by the authors was used for the choice of material parameters and particle size distribution. In [24], the crushing of two different types of rail sand was simulated in single grain crushing tests. The rail sands were named GB and AT according to their applications in Great Britain and Austria. In this work, the same sand types will be used as granular material with the material parameters from [24]. Table 2 gives the material parameters for both sand types and steel (surface spheres). In [24], single grain crushing tests under a realistic wheel–rail load of 900 MPa were simulated. The granular material used in this work had the same particle size distribution obtained from the final state of these simulations, see Fig. 7 for GB sand.

**Table 2:** Parameters of Hertz–Mindlin contact law for considered materials: AT and GB sand

material	*E* [GPa]	$\nu$ [-]	$\mu$ [-]	$\rho \left[ \frac{\text{ kg }}{\text{ m}^3}\right]$	$e_n$ [-]
GB sand	86.5	0.3	0.5	2650	0.5
AT sand	79.1	0.3	0.7	2650	0.3
steel	200.0	0.28	0.4; 0.2	7833	

**Fig. 8:**
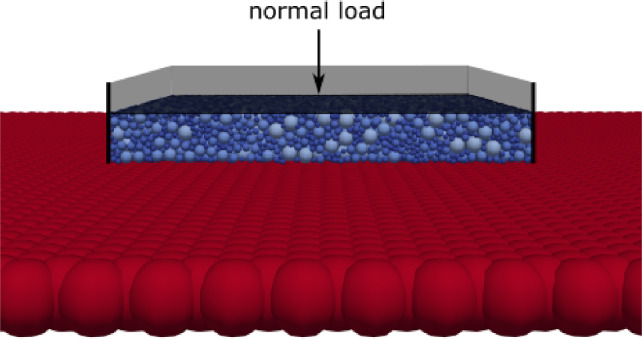
Setup of granular compression on surface grid

**Fig. 9:**
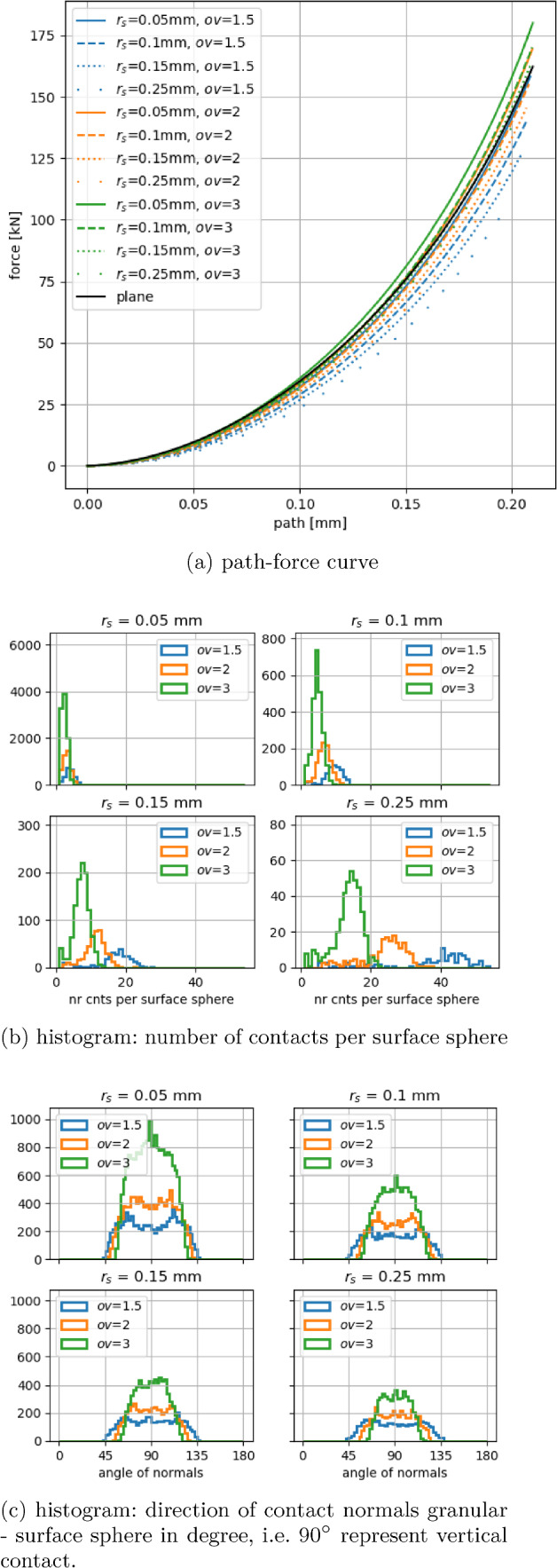
Elastic compression test: granular material on surface grid and ideal plane

In the chosen setting, the granular material is compacted in a box of 3 mm $\times$ 3 mm side length, see Fig. 8. This is the approximate size of a cluster of solidified sand fragments forming in compression tests of GB sand. The mass of one initial sand grain of GB sand is filled in the box with a plane bottom, which results in a number of approx. 15,000 spheres representing the sand grain fragments. The grains are generated in a loose cloud above the box and are left to settle under gravity using a reduced coefficient of friction of $\mu _r=0.01$. Afterwards, the sample is compacted by a plane until 10N and is left to settle again. In the simulated compression tests, the generated granular sample is located above a surface grid and then compacted by a plane until a loading path of 0.22 mm was reached. The resulting path-force curves for the 12 surface grids used can be seen in Fig. 9a together with the simulation result belonging to a bottom plane. In contrast to the setting of the elastic indentation of a large sphere, here only a moderate difference between the results of a bottom plane and the surface grids can be seen. The reason for this behaviour is related to the equivalent radius used in normal force calculation in Hertzian theory, $\frac{1}{R^*}=\frac{1}{R_1} + \frac{1}{R_2}$. When the granular spheres contact the bottom plane, the equivalent radius is equal to the granular sphere’s radius. When a granular sphere contacts a larger surface sphere, the equivalent radius approximately equals the smaller granular sphere’s radius. Deviations are caused by cases of granular spheres being of similar size as surface spheres and cases, where a granular sphere is in contact with more than one surface sphere. In Fig. 9a, the lowest resulting force belongs to the largest surface spheres and the lowest overlap, i.e. $r_s=0.25~\hbox {mm}$, $ov=1.5$. With both decreasing $r_s$ and increasing *ov*, the number of surface spheres in contact with the granulate increases, which leads to higher resulting forces. Thus, the highest resulting force belongs to $r_s=0.05~\hbox {mm}$, $ov=3$.

The contact between the surface spheres and the granular material is further characterised in Fig. 9. In Fig. 9b, histograms are shown of the number of contacts per surface sphere. The four subplots contain the histograms of the three considered overlap values *ov* for each considered surface sphere radius $r_s$. As expected, higher overlap values result in lower numbers of contacts per surface sphere, and larger values of $r_s$ result in higher numbers of contacts per surface sphere. The peak values in the histograms belonging to $r_s=0.05~\hbox {mm}$ are 3, 3, and 2 for the overlap values $ov=1.5, 2, 3$. For the largest radius considered, $r_s=0.25~\hbox {mm}$, the corresponding peak values were 40, 26 and 14 for $ov=1.5, 2, 3$, respectively.

**Fig. 10:**
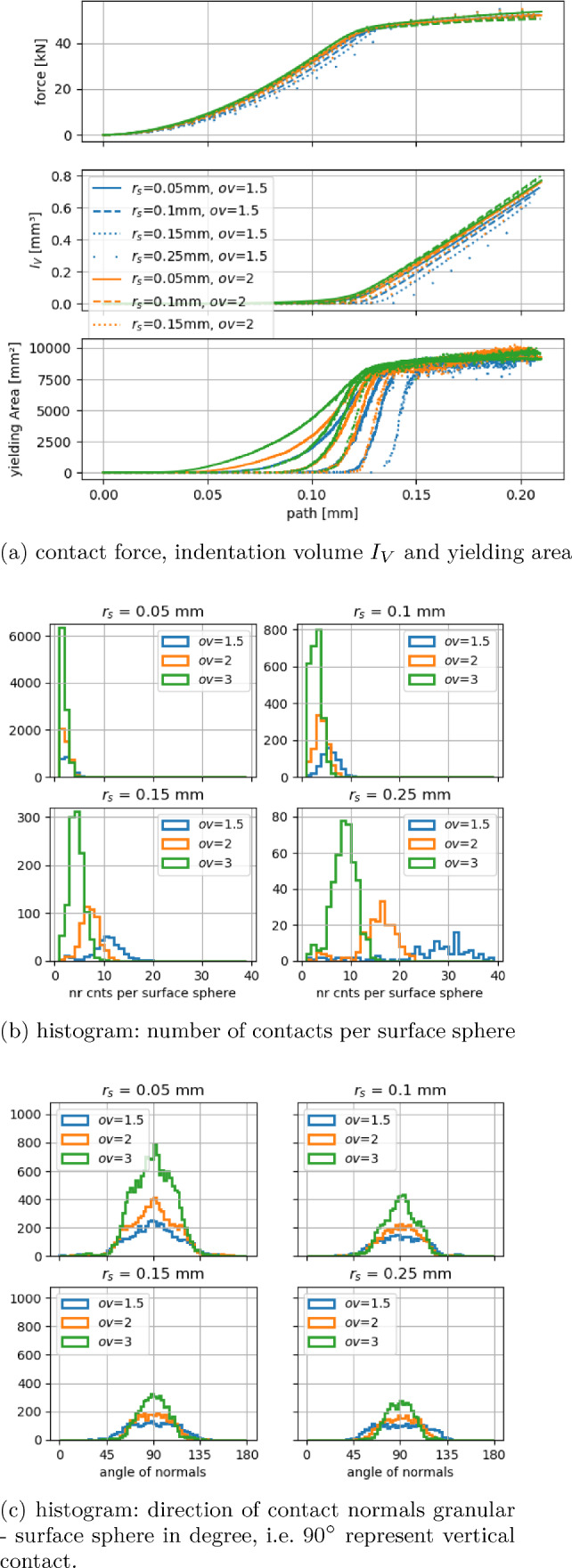
Plastic compression test: granular material on surface grid

Furthermore, the normal direction of all contacts between surface spheres and granular material is studied in Fig. 9c. Here, histograms of these normal directions are shown, where a vertical contact is represented by $90^\circ$. Expectedly, the highest overlap value and the smallest radius give the smoothest surface representation and thus the narrowest histogram with a pronounced peak at $90^\circ$. With reducing *ov* and increasing $r_s$ the modelled surface is rougher, which leads to broader histograms with a plateau instead of a peak at $90^\circ$. In the elastic case, the minimal/maximal possible angles are limited by the grid’s opening angle, $\phi$, which depends only on the overlap *ov*.

### Plastic normal compression of granular material

Next, the same granular compression test is considered, where the surface grid is allowed to deform plastically. The hardness $H=5~\hbox {GPa}$ was chosen for the simulations of these tests.

Figure 10a shows the path-force curves belonging to the 12 surface grids as well as the development of the indentation volume $I_V$ and the yielding area $A_y(t)=\sum _{j=1}^N y^i_V(t) \,a_s$. At first, the contacts of granulate and surface spheres are mostly in the elastic range, with some yielding contacts. In this phase, differences in the resulting force develop between the grids, as it was seen in the previous subsection. The amount of yielding contacts can be seen best from the yielding area. It shows huge differences between the grids, due to the different elastic response and the flow condition influence discussed in Sec. 3.3, $F(r_s) > a_s(r_s, ov) H$. As a result, the grid of smallest spheres with the largest overlaps ($r_s=0.05~\hbox {mm}$, $ov=3$) reaches the phase of almost pure yielding before the grid of largest spheres with the smallest overlaps ($r_s=0.25~\hbox {mm}$, $ov=1.5$) shows severe yielding at all. In this last phase of mostly yielding contacts, a massive increase in the indentation volume $I_V$ is seen, which is enforced by the external load and thus similar for all surface grids.

In Fig. 10, the analogous plots as in the purely elastic case are shown. The histograms of the number of contacts per surface sphere, shown in Fig. 10b, have their peaks shifted towards smaller contact numbers. For $r_s=0.25~\hbox {mm}$, these peak values are 32, 17, 9 (elastic case: 40, 26 and 14) for the overlap values $ov=1.5, 2, 3$, respectively. Also, these peaks show a higher count compared to the elastic case, while the overall number of contacts between granulate and surface spheres decreased compared to the elastic case. Thus, due to the plastic deformation of the surface grid, contacts with the granulate are reduced, and more of the surface spheres have a lower number of contacts with the granulate compared to the purely elastic case.

Regarding the contact normal directions shown in Fig. 10c, the effect of the plastic deformation is harder to quantify. Overall, the shape of the histograms is more peaked around the vertical contact of $90^\circ$. However, there are a few contacts with normals below $45^\circ$ or above $135^\circ$. They belong to surface spheres at the edge of the box, where the spheres are only partially yielding. These cases did not occur in the elastic case, where the minimum and maximum angles in Fig. 9c were limited by the surface grid’s opening angle $\phi$.

Finally, a brief comparison of the computational times of these simulations is made. All simulations run on an Intel(R) Xeon(R) Gold 6254 CPU @ 3.10GHz processor. For a fixed grid sphere radius $r_s$, the number of grid spheres also increases with increasing overlap *ov*, compare the upper subplot of Fig. 4. For grids with the largest grid sphere radius, $r_s=0.25~\hbox {mm}$, the number of grid spheres lies between roughly 900 and 4,000, and the computational times ranged between 17 and 20 h. For grids with the smallest grid sphere radius, $r_s=0.05~\hbox {mm}$, the number of grid spheres lies between roughly 25,000 and 100,000, and the computational times ranged between 23 and 28 h. While the differences in computational time are clearly dominated by the number of grid spheres, the onset of yielding contacts also plays a role, which differs for the different grids, see the description above.

### Summary of grid influence

After the analysis of the surface grids’ influence on the simulated test cases, it has to be decided, which grid should be used. Due to several advantageous properties, the grid ($r_s=0.15~\hbox {mm}$, ov= 2) is considered from now on. With 4810 surface spheres, it combines a moderate computational effort with a suitably smooth surface representation regarding hd and $\phi$, see Fig. 4. Moreover, the chosen approach aims at describing the surface as a continuum, which means that each surface sphere should have several contacts with the granulate. This demand is met, compare Fig. 10b, and the chosen grid also shows a moderate yielding behaviour, compare the lowest subplot of Fig. 10a.

**Fig. 11:**
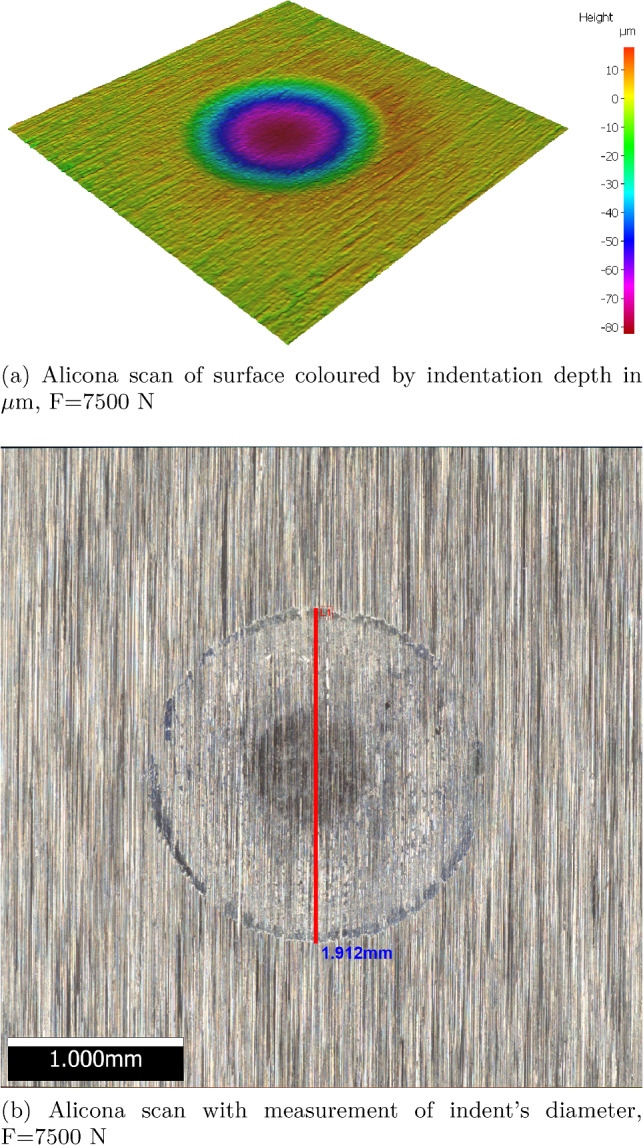
Spherical indentation tests: Alicona scans of indentation depth and indent’s diameter

**Fig. 12:**
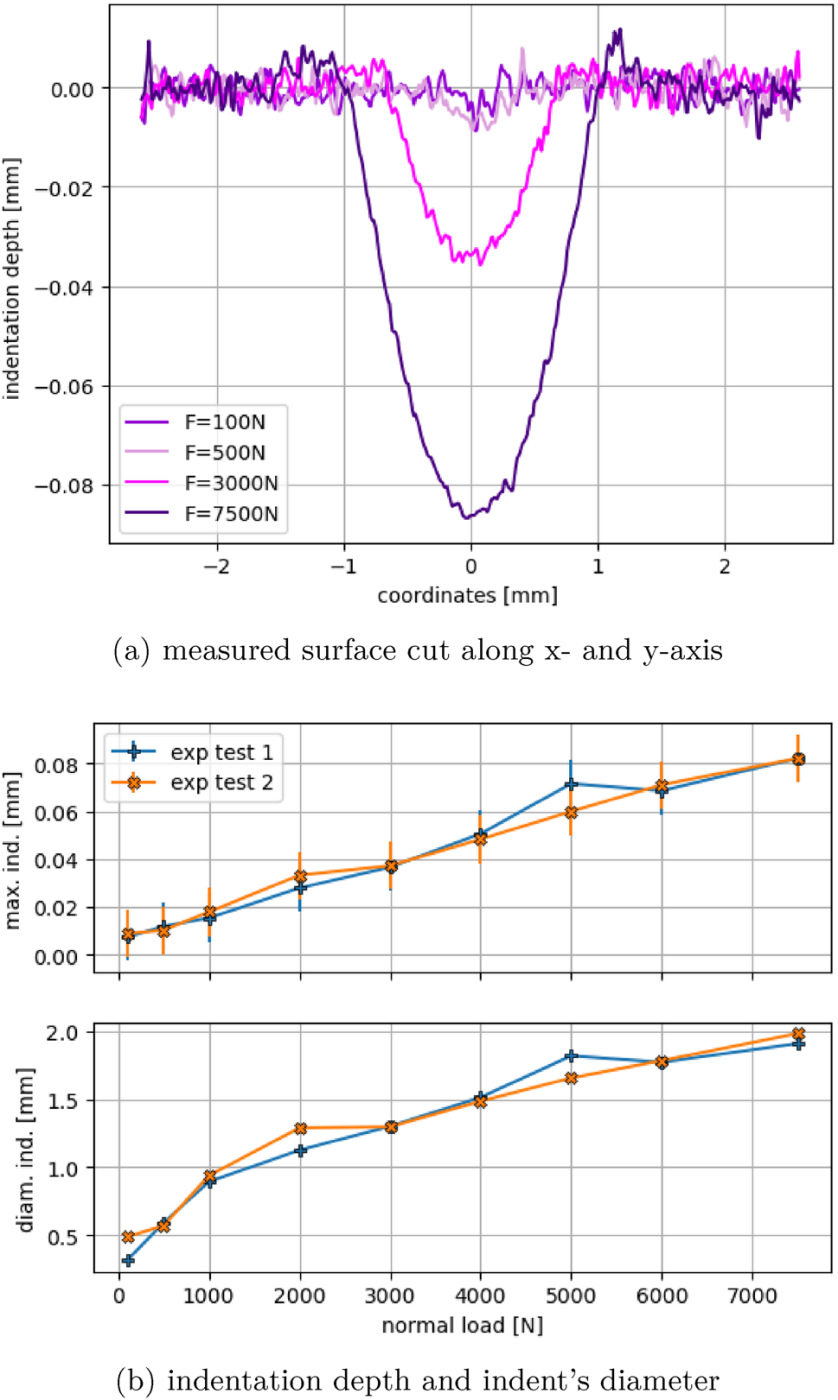
Spherical indentation tests: examples of cut through measured point cloud and maximal indentation and indent’s diameter for all load levels

## Experimental indentation tests and parametrisation

For the parametrisation of the hardness *H* in the developed DEM model, spherical indentation tests were conducted on a typical rail steel called R260. These tests were conducted using a servo-hydraulic test machine. A flat specimen made of rail steel was placed into a bottom holder with a stainless steel (AISI 440C) ball bearing of 8.73 mm diameter, fixed into a top holder. The flat specimen and ball bearing were brought into contact, and normal loads were applied at the following levels: 100, 500, 1000, 2000, 3000, 4000, 5000, 6000, 7500 N. For each load level, one repetition was conducted to check the quality of the measurement.

After the indentation test was complete, the indent was analysed using an Alicona InfiniteFocusSL 3D optical profilometer. The Alicona captured a 3D scan covering a 3.66 mm $\times$ 3.66 mm area (vertical resolution of 500 nm). Further post-processing of the image conducted using in-built software yielded an image coloured by the indentation depth, see Fig 11a for an example. Also, a picture of the indent used to visually measure the intent’s diameter was taken, see Fig 11b for an example. Finally, with the Alicona a.txt file of the measured point cloud of the indented surface was saved. These point clouds were centred around 0 and rotated such that the indentation was parallel to the *z*-axis. Cutting through the post-processed point clouds allowed visualisation of the indent’s shape in 2D, see Fig. 12a for some examples. Here, the surface roughness can be seen at the unindented area, i.e. the edge area of the steel plate. In this area, $R_q$ values were calculated, which had a median value of $2.8~\mu \hbox {m}$. In this area, the *z* values lay mostly between $\pm 10 \mu \hbox {m}$. The indentation depth was calculated as the minimum *z* value in the neighbourhood of 0. The calculated indentation depth and the visually measured indent’s diameter are shown in Fig. 12b for all load levels. The indentation depth plot shows error bars with the mentioned $\pm 10 \mu \hbox {m}$. This is especially important for the two lowest load levels, as for 100 N nearly no indent and for 500 N very small indentation depth is calculated. A linear relationship between the applied normal load and indentation depth can be seen. The agreement of the two measurements taken at each load level is high, with the exception of test 2 at 2000 N and test 1 at 5000 N, see Fig. 12b. For high loads in Fig. 12a, a slight lateral flow of the material can be seen, i.e. material is accumulating at the edge of the indent. It should be noted that this effect is not included in the DEM model.

In addition to investigating the indented steel surface, also scans were taken of the ball bearing intermittently to ensure large-scale plastic deformation was not occurring. The generated measurement data are freely available at zenodo.org, see [34].

**Fig. 13:**
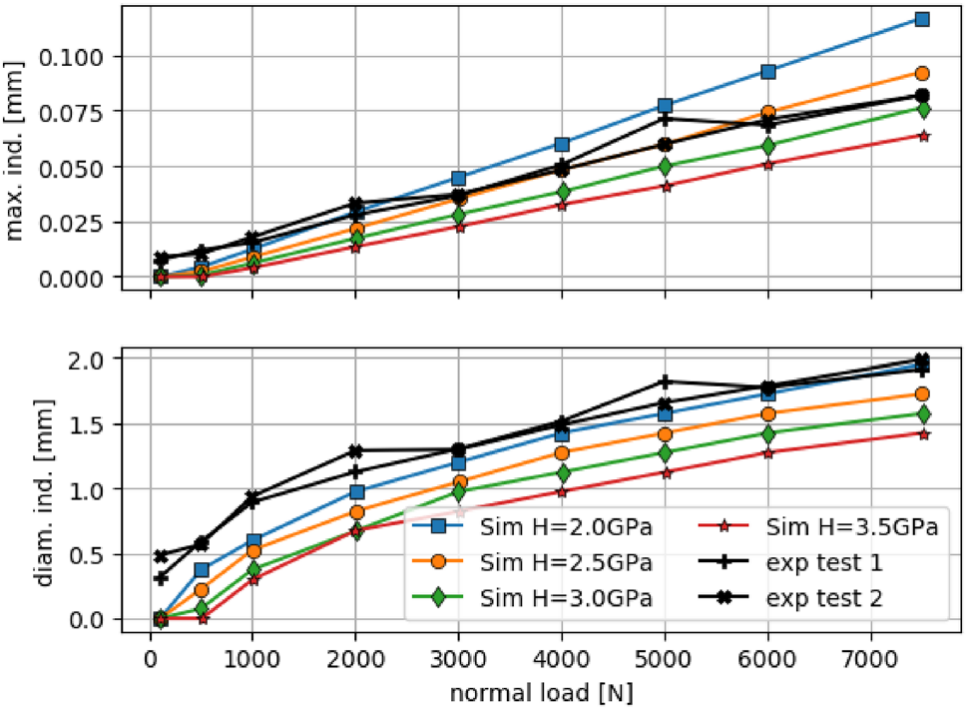
Spherical indentation tests: Comparison of experimental and simulation results

**Fig. 14:**
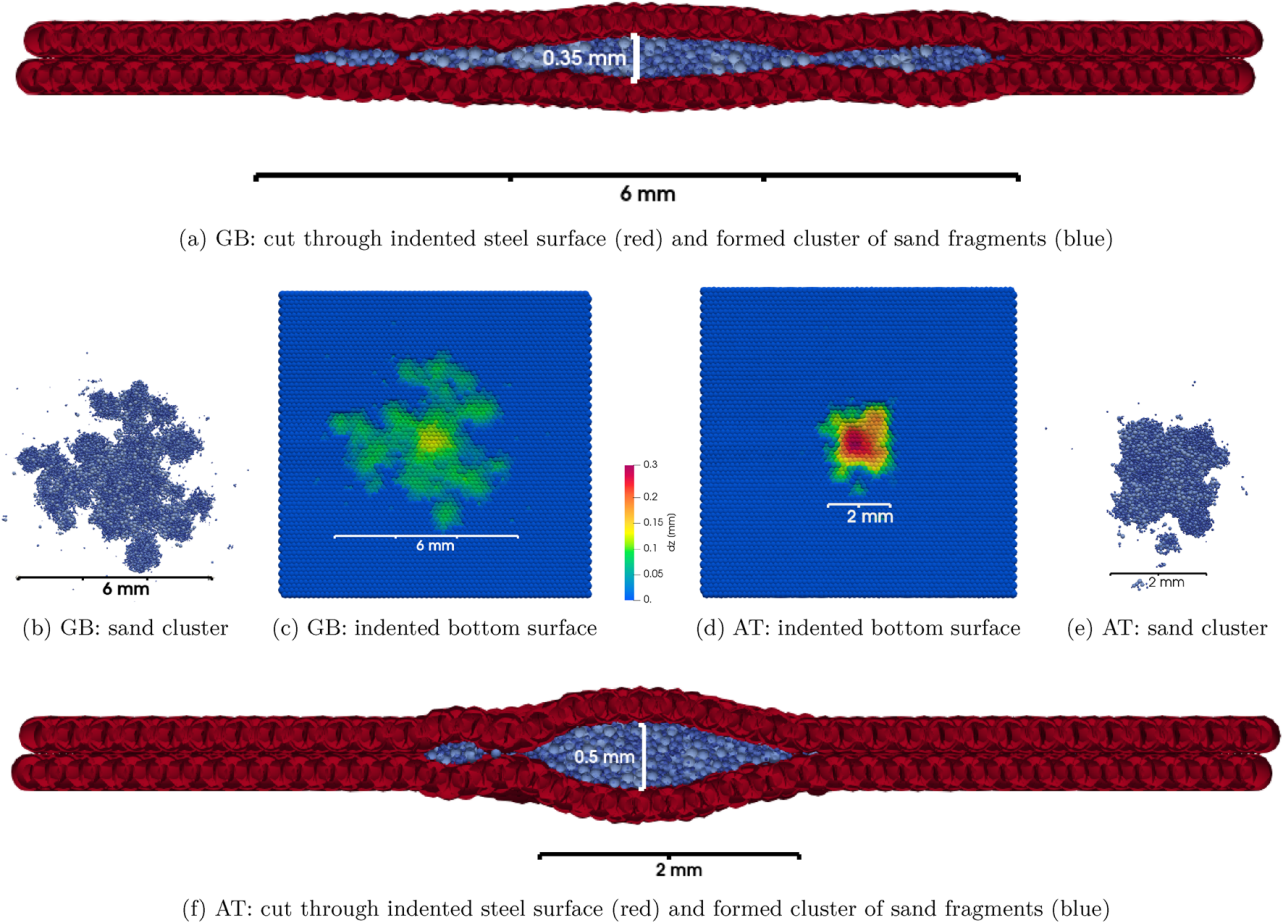
Single sand grain crushing for GB and AT sand with indented steel plates of $H=2.5~\hbox {GPa}$

For the parametrisation of the DEM model, simulations of the indentation tests were conducted, using different values for the hardness *H*. The used surface grid is ($r_s=0.15~\hbox {mm}$, $ov= 2$), and the material parameters for the steel surface and the indenter sphere are the same as given in Table 2. In the simulations, the steel surface is not moving, while the indenter sphere moves with constant velocity. When a load level is reached, the maximal plastic indentation of the surface grid and the diameter of the formed indent at the surface grid are saved. The load is further increased until the highest load level is reached. These simulations are conducted with several hardness values *H* and their results are compared with the experimental measurements at each load level, see Fig. 13.

The measured maximal indentation depth was in good agreement with simulations using $H=2.5~\hbox {GPa}$, especially for loads above 2000 N. With increasing hardness values, the simulated surface starts to deform at higher loads. In all cases, the same linear relationship between applied load and indentation depth as in the experimental results can be seen. Due to the good agreement between measured indentation depth and the results of the elastic-(ideal)plastic simulation model, it seems that hardening of the steel does not play a role in these tests. Considering the indent’s diameter, simulations with $H=2~\hbox {GPa}$ showed the highest agreement with the experiments. As the diameter was measured visually from the photo taken by the Alicona, these values were considered less precise. Therefore, the steel surface of R260 rail steel will be simulated using $H=2.5~\hbox {GPa}$.

## Proof of concept: application to wheel–rail sanding

The aim of the work presented here is to do one step towards the development of a DEM model for wheel–rail sanding. In previous works [23], a single sand grain was crushed between two hardened steel plates under a realistic wheel–rail load of 900 MPa. Two types of rail sand were investigated, and two contact conditions: dry and wet contact. Under wet contact conditions, both types of sand formed clusters of solidified sand. Although hardened steel plates were used to minimise plastic deformation, after testing, indents of approx. $40\mu \hbox {m}$ depth were seen in Alicona scans. In [24], a DEM model for these single grain crushing tests was developed, where the steel plates were considered undeformable. A good qualitative agreement between experiments and simulations could be achieved regarding the formation and size of clusters of solidified sand. However, there was a problem with very high overlaps of sand fragments, which was caused by the extremely high applied stress and the rigid steel plates used in these simulations.

In this section, the model developed in [24] will be combined with the surface indentation model developed in this work. As no experimental data are available yet for direct comparison, this effort is seen as a proof of concept.

Single grain crushing tests of GB and AT sand under 900 MPa load are simulated under wet contact conditions. The full details of the method can be found in [24]. The material parameters for sand and steel for the Hertz–Mindlin contact law are given in Table 2, and the parameters of the sand breakage model, including cohesion, are chosen as in [24]. The steel surfaces are modelled with the surface grid ($r_s=0.15~\hbox {mm}$, $ov= 2$), and the parametrised hardness $H=2.5~\hbox {GPa}$ is used. During the simulation, the sand fragments fracture repeatedly and form clusters of solidified sand. The steel plates remain undeformed while the majority of breakage events take place. For both types of sand, the plastic indentation of the steel surface does not start before more than 95% of all sand fragments have a size below the breakage limit and are thus considered unbreakable. Only in the last part of the simulation, the occurring stresses are high enough to indent the steel plates. The final state of the simulations for GB and AT sand can be seen in Fig. 14. In Fig. 14a, a cut through the indented steel plates enclosing the formed sand cluster is shown for GB sand. Figure 14b and c shows a top view of the formed sand cluster and the indented steel surface, coloured by the indentation depth, also for GB sand. The analogous figures for simulations of AT sand can be seen in Fig. 14d, e and f, respectively. In simulations of both sand types, at the final state there is contact, and thus load transfer, between the two steel plates. At 1.54 mm, the initial diameter of the GB sand grain is larger than the one of the AT grain at 1.25 mm. The size and height of the formed cluster differ between the sand types, which leads to different maximal indentation depths and indentation volumes of the steel surfaces. For GB sand, the formed cluster has an approximate length of 5 mm, the maximal indentation depth is 0.16 mm and the indentation volume of the bottom steel plate is $1.25~\hbox {mm}^3$. For the AT sand, the formed cluster has a length of approximately 2 mm and the maximal indentation depth is at 0.23 mm higher than for GB sand. Due to the smaller initial grain size of AT sand, the indentation volume of the bottom steel plate is $0.71~\hbox {mm}^3$ and thus lower than for GB sand.

As mentioned above, the model developed in [24] using rigid steel surfaces suffered from very high particle overlaps. These high overlaps are now considerably reduced. For quantification, the overlap between two particle’s is divided by the smaller particles radius, called overlap ratio, i.e. at a value of 2.0 the smaller particle would be completely enclosed by its contact partner. In a simulation of GB sand without the surface indentation model, 80% of all contacts have overlap ratios below 0.45. This value reduces to 0.3 in the simulation with the surface indentation model. For simulations of AT sand, the overlap ratios are even higher (as for the smaller initial grain, local stresses are higher). The value is 0.73 for rigid steel surfaces and reduces to 0.34 for the simulation with the surface indentation model. Still, these overlap ratios are still higher than in usual DEM simulations taking into account elastic material behaviour. This is to be expected, as under the extremely high stresses, the material behaviour is much more complex, e.g. formation of solidified clusters. The used elastic contact model with the very high cohesion values is seen as a substitute until physical effects are understood better and allow for a more detailed modelling.

The simulated indentation depth is clearly higher than the 0.04 mm seen in the experiments conducted in [23]. However, these experiments were conducted with hardened steel to minimise indentations on the surfaces as much as possible. As a comparison, hardness measurements of the hardened steel and the R260 rail steel used in this work were taken. Taking the average from five measurements per steel type, the hardened steel has a hardness of 765 Hv, while the R260 steel has a hardness of 316 Hv. Taking the ratio of these values, 765/316=2.42, and assuming a linear relation between the measured hardness and the model parameter *H*, gives approximately $H=6~\hbox {GPa}$ for the hardened steel plates. Again, simulations for AT and GB sand are conducted with $H=6~\hbox {GPa}$. At the final state, the maximal indentation depth is 0.08 mm for GB sand and 0.19 mm for AT sand. In the simulations of GB sand, the steel plates are not in contact, thus the load is solely transferred through the sand cluster. This can be related to the larger surface area of the cluster, thus causing lower local stresses when compared to AT sand. The simulated maximal indentation depths are still higher than the experimentally observed values. Most likely, the assumed linear scaling between measured Hv values and the model parameter *H* could be inappropriate. However, differences between simulations and experiments could also originate from physical mechanisms not included in the model or general model deficiencies.

## Conclusions and outlook

This work deals with DEM modelling of a surface indented by a granular material. The application of this topic is wheel–rail sanding. This process is frequently used in railways to overcome low-adhesion conditions, while the physical mechanism of adhesion increase is not well understood. A DEM model can help to deepen this understanding. As a preparation, in [23], single sand grain breakage tests under realistic wheel–rail load, 900 MPa, were conducted on two types of rail sand under dry and wet contact conditions. The sand grains fractured repeatedly and, dependent on the sand type and contact condition, formed clusters of solidified sand fragments. These clusters indented the hardened steel plates used in the tests. In [24], a DEM model of these tests was developed and parametrised. The steel plates in the crushing tests were modelled as undeformable in [24].

This work presents the next step, where a solid’s surface can be indented by granular material. An existing surface indentation from the literature [2], was adapted for indentations by a granular material under normal load. The indented surface is modelled in DEM as a regular hexagonal grid of spheres, and the influence of grid discretisation is investigated in two test cases: normal impact of a spherical indenter on the surface and normal compression of a granular material on the surface. After the optimal grid is chosen, the DEM model is parametrised using spherical indentation tests on a typical rail steel R260. Simulation results are in good accordance with the indentation depth calculated from measurement data.

In a proof of concept, the single sand grain crushing tests under realistic wheel–rail load from [24] are combined with the new surface indentation model. Simulations of GB and AT sand show differences in the size of formed clusters, maximal indentation depth, and indented volume. No measurement data are available for a direct comparison of the simulated indentation of the steel plates. The model of [24] using rigid steel surfaces had a problem with very high particle overlaps. Combining [24] with the surface indentation model considerably reduces the observed overlaps.

The obtained results are an important step on the way of building a DEM model of wheel–rail sanding. Planned next steps are to study the shearing behaviour of the two types of rail sand in a small-scale shear box test.

## Data Availability

The datasets analysed during the current study are openly available in the zenodo.org repository, see [34].
